# CSPP1 preserves quiescent microtubule functions by dual-end capping

**DOI:** 10.1093/jmcb/mjae022

**Published:** 2024-05-21

**Authors:** Marina Mapelli

**Affiliations:** European Institute of Oncology IRCCS, Via Adamello 16, 20139 Milano, Italy

Microtubules (MTs) are cytoskeletal filaments composed of polarized arrays of globular α/β-tubulin dimers organized as hollow cylinders. Thanks to this arrangement, MTs are mechanically rigid and can assemble into large intracellular structures that serve diverse cellular functions. In dividing cells, MTs organize bipolar mitotic spindles, ensuring equal segregation of sister chromatids and correct placement of daughter cells into the tissue (Gudimchuk and McIntosh, 2021; Lechler and Mapelli, 2021). In interphase and post-mitotic cells, MTs sustain nuclear positioning, intracellular transport, and the crosstalk with the actin cytoskeleton to promote cell motility and morphological changes. These functionalities are enabled by dynamic instability of the MT filaments at both ends, which continuously switch between the phases of growth and shrinkage assisted by specialized MT-associated proteins (MAPs) (Gudimchuk and McIntosh, 2021). In a recent article published in *Journal of Molecular Cell Biology*, Wang et al. (2024) described the dual-end capping activity of centrosome and spindle pole protein 1 (CSPP1), adding an extra layer to the known repertoire of MAPs that control MT-dependent cellular functions.

MTs dynamically interact with cellular components to control fundamental processes, and defective MT functions are implicated in many human pathological conditions. Therefore, understanding the working principles of MAPs is of great interest. Previous studies already generated an extended knowledge of how individual MAPs spatiotemporally stabilize or destabilize MTs by targeting soluble α/β-tubulin dimers, the MT lattice, and MT ends. Among MT end-binders (EBs), plus-end tracking proteins (+TIPs) accumulate at growing plus-ends to prevent catastrophe and sustain elongation, while minus-end capping proteins arrest both polymerization and depolymerization to favor the formation of MT-organizing centers. To date, the best characterized minus-end cappers are γ-TuRC and CAMSAPs (Akhmanova and Kapitein, 2022). In addition, in cellular structures harboring stable MTs, including neuronal axons and ciliary structures, stabilizing proteins can be found in the inner MT lumen (Szyk et al., 2014). However, less is known about how non-centrosomal MTs maintain their quiescent state at plus- and minus-ends when dynamic instability is not needed.

CSPP1 is an MT-binding protein localized to spindle poles and central spindles in mitosis, to centriolar satellites in interphase, and to ciliary axonemes. Loss-of-function mutations of CSPP1 lead to ciliopathies, including Joubert syndrome (Asiedu et al., 2009; Akizu et al., 2014; Shaheen et al., 2014). The mechanistic basis for its function has remained unclear until recently. By live-cell imaging of epithelial cells, Wang et al. (2024) demonstrated that CSPP1 is localized at both minus-ends and the EB3-decorated plus-ends of non-centrosomal MTs. Notably, the enrichment of CSPP1 at MT ends was enhanced after MT laser ablation, suggesting that CSPP1 helps maintain MT integrity. To better understand the molecular basis of the MT-binding activity of CSPP1, the authors expressed different CSPP1 domains in CSPP1-depleted cells and quantified MT dynamic instability. These experiments revealed that CSPP1 acts as an MT stabilizer by prolonging the pausing time and reducing the growth speed, which is entirely dependent on its central MT-binding domain. Kymographs of quantitative total internal reflection fluorescence (TIRF) microscopy conducted *in vitro* with reconstituted MTs and purified CSPP1 revealed that CSPP1 directly alters dynamic instability by abrogating MT catastrophe and decreasing growth rates at both MT ends, and such effect is recapitulated to a great extent by its MT-binding domain.

The extensive interaction network of +TIPs includes members of the kinesin-13 family, such as mitotic centromere-associated kinesin (MCAK) that binds to EB1 and depolymerizes MTs by removing α/β-tubulin dimers, thereby promoting continuous MT pruning (Tanenbaum et al., 2011). To explore potential crosstalk between CSPP1 and MCAK, the authors performed TIRF experiments in which they incubated MCAK with MTs in the presence or absence of sub-stoichiometric amounts of CSPP1 or its MT-binding domain. These experiments demonstrated that CSPP1 protects MTs from MCAK-mediated shortening.

**Figure 1:**
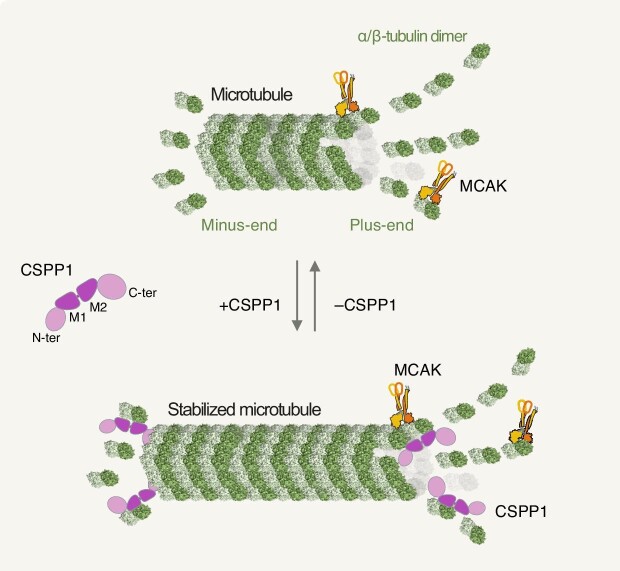
The plus- and minus-end capping protein CSPP1 stabilizes MTs. Top: MTs are formed by polar filaments of α/β-tubulin hetero-dimers (in light and dark green) arranged as hollow cylinders. Dynamic instability leads to the shortening of non-centrosomal MTs by shrinking and depolymerization, which is assisted by the kinesin-13 family member MCAK (homo-dimer in gold and orange) accumulating at plus-ends. Middle left: CSPP1 consists of an N-terminal domain, two central MT-binding domains (M1 and M2), and a C-terminal region. Bottom: CSPP1 preferentially associates with MT ends via its MT-binding domains. The binding of CSPP1 to MT ends prevents pruning, counterbalancing the activity of MCAK.

Collectively, the findings from Wang et al. (2024) uncover a novel dual-end capping activity of CSPP1 that preserves the length of quiescent MTs, antagonizing the MCAK destabilizing function (Figure 1). These findings are corroborated by a study by van den Berg et al. (2023) showing that CSPP1 exerts its MT stabilizing effect by localizing to the inner MT lumen. These discoveries enlarge the landscape of MT regulators orchestrating biological functions in non-mitotic cells where stable MT structures are present. Further studies will be required to understand how CSPP1 interacts with other MT-end binders and how its activity is regulated in a context-specific manner. Considering the crucial role of CSPP1 in ciliopathies, investigating its cellular activity and understanding its physiological implications hold great promise. This helps unveil unexplored layers of multicellular organism development and eventually paves the way for developing novel therapeutic strategies to cure ciliopathies.
